# Exploring trends in the ambulatory nursing practice environment and nurses' career intentions: 2021 to 2023

**DOI:** 10.1097/nmg.0000000000000289

**Published:** 2025-09-02

**Authors:** Deb Cantlin, Angela Pascale, Elizabeth Fritz, Margo A. Halm, Brenda Luther, Nora E. Warshawsky

**Affiliations:** **Deb Cantlin** is a nurse educator at Dartmouth-Hitchcock Medical Center in Lebanon, NH. **Angela Pascale** is a research analyst at Press Ganey Associates in Chicago, IL. **Elizabeth Fritz** is an RN-scientist at SSM Health in St. Louis, MO. **Margo A. Halm** is a nurse scientist at the American Academy of Ambulatory Care Nursing in Pittman, NJ. **Brenda Luther** is a professor at the University of Utah, College of Nursing in Salt Lake City, UT. **Nora E. Warshawsky** is a nurse scientist at Press Ganey Associates in Chicago, IL.

**Figure:**
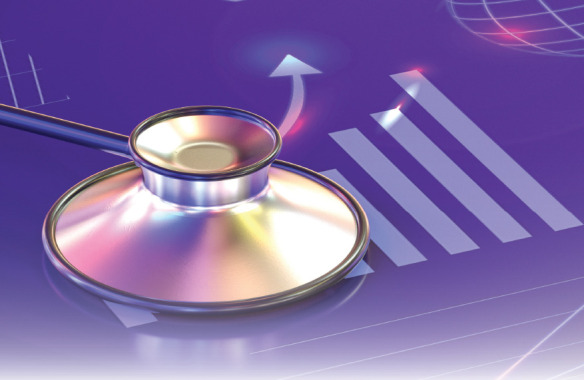
No caption available.

According to the 2024 NSI National Healthcare Retention and RN Staffing Report, hospital administrators and nurse leaders are concerned about the cost of registered nurse (RN) turnover, as well as the direct effect of fewer RNs on patient safety and quality.^1^ The average cost of turnover for a direct care nurse is estimated at $56,300.^1^ Nurses are also concerned about why colleagues choose to stay or leave the profession. Understanding the variables affecting the nursing shortage is crucial. What are the factors affecting nurses' decision to leave the profession? What factors are within our control?

Nurses have identified various factors that contribute to nurse retention, including practice autonomy, workload, role clarity, and organizational and structural support for nurses to govern their own practice while providing essential care. Common burnout risk factors include high workload, insufficient support and/or resources, work-life imbalance, lack of autonomy, and challenges within the organizational climate.^2^ Although there are many environmental aspects that contribute to nurse burnout and retention, we surmise each plays a role in overall nurse satisfaction.^3^,^4^ Understanding factors that mitigate nursing burnout is vital to ensure the ongoing health and well-being of the nursing workforce. Nurse surveys are one strategy to identify discrete, real-time data related to intent to leave and/or stay in the profession.

## BACKGROUND

Nursing practice has expanded beyond that of a direct care nurse for hospitalized patients. The bulk of care is currently provided in ambulatory environments. Up to 23 times more people receive medical care in office-based or outpatient settings than in hospitals.^3^ Patients are discharged sooner than they used to be and have continued acute and chronic needs. Ambulatory care nurses fill various roles in clinics, stand-alone infusion centers, surgery centers, correctional facilities, public and private schools, and home care. Ambulatory care work environments vary significantly from acute care environments. A recent study identified several features of the ambulatory care environment that influenced how work was conducted in this setting.^5^ These features included having a large geographic footprint because many nurses work at multiple clinic locations; delivering patient care focused on wellness and the prevention and management of chronic illness; and navigating varying regulatory requirements and specialty accreditations, depending on the clinic.

Recent literature has identified characteristics of ambulatory care work environments that can influence how nurses work and how satisfied they are with their work. Brzozowski and colleagues noted many ambulatory care nurses have opportunities to work in primarily telephonic or virtual encounters.^6^ Much of their work involves helping patients navigate the healthcare system and manage healthcare transitions. Brzozowski and colleagues also found that differences in clinic populations (for example, pediatric and geriatric) and physical layout (such as pods) influenced how nurses practiced in those settings.^7^ Another article identified lack of prelicensure exposure to ambulatory care as a contributor to challenges for new ambulatory care nurses acclimating to their setting.^8^

In a systematic review of contributors to nursing burnout in ambulatory care, Lieneck and colleagues identified a combination of homelife stress, lack of outpatient facilities, lack of organization or administration support, and work-related issues such as short staffing as key factors in ambulatory care nurse burnout.^4^ This review noted that high workloads and inadequate staffing and equipment are important environmental characteristics to address. Brzozowski and colleagues identified ways in which primary care work settings influenced nurses' perceptions of the work environment.^7^ Primary care nurses working in Magnet® organizations, when compared with nurses in non-Magnet organizations, rated their leaders higher and felt that their personal values were more aligned with their leaders' values. These findings were consistent with previous literature linking nurse satisfaction with the indicators of nursing excellence used by Magnet.^9^

## SPECIFIC AIM

This study's aim was to describe national trends in the ambulatory care nursing practice environment and the career intentions of clinic-based ambulatory care nurses from 2021 to 2023. By understanding the core components of this unique environment and nurses' career intentions, ambulatory care nurses and their leaders can collaborate to implement strategies to improve aspects of the work environment to retain and recruit nurses.

## METHODS

### Design

This study utilized data from the annual National Database of Nursing Quality Indicators® (NDNQI®) RN Survey conducted in 2021, 2022, and 2023, including all ambulatory care units and direct care nurses who participated during at least one of those years. Press Ganey client hospitals voluntarily participated in the RN survey to collect data on nurse outcomes, work environment, and individual RN characteristics. Clinic-level data were analyzed to understand trends in the work environment and nurses' short- and long-term employment intentions in ambulatory care settings.

### Sample

RNs were eligible to participate in the NDNQI RN Survey if they had been employed for a minimum of 3 months in their current unit and spent at least 50% of their time providing direct patient care. Ambulatory care units included cancer care, cardiac services, endoscopy, interventional cardiology/radiology, outpatient clinics, primary care, procedural, short stay/observation, specialty care, and urgent care.

### Measures

***Practice Environment Scale (PES)***. Work environment was measured using the PES of the Nursing Work Index (PES-NWI).^10^ The PES scale assesses nurse perceptions of their work environment with 31 items across five subscales: Nurse Participation in Hospital Affairs; Nursing Foundations for Quality of Care; Nurse Manager Ability, Leadership, and Support of Nurses; Staffing Resources and Adequacy; and Collegial Nurse-Physician Relations.Each item is rated on a 4-point Likert-type scale ranging from 1 (strongly disagree) to 4 (strongly agree), with higher scores reflecting more positive perceptions of the practice environment. The PES subscales and composite have demonstrated strong reliability (α > 0.70) and construct validity, supported by significant associations with nurse-reported outcomes and patient outcomes.^10^,^11^

***Job plans and intent to leave***. Job plans were assessed using an item from the NDNQI RN Survey asking RNs to report their job plans for the next year and the next 3 years. RNs were provided with the following three response options: plan to stay in my current position and work setting, plan to stay in nursing but change current position and/or work setting, and plan to leave nursing. Those who selected that they planned to leave nursing or to stay in nursing but change their current position and/or work setting were then asked to select from a list of reasons driving their intent. Within the NDNQI survey, reasons for leaving are categorized as either change in nursing career, work-related dissatisfaction, or home and personal life.

### Data analysis

Descriptive statistics (mean/standard deviation for continuous measures and frequencies/counts for categorical measures) were used to summarize annual PES scores and trends in job plan distributions from 2021 to 2023. For work environment, units with fewer than five RN responses in a given survey year were excluded from this portion of the analysis. Unit-level mean PES scores and mean subscale scores were calculated for each year of the study period. For the individual RN-level analysis of job plans, all direct care nurses from ambulatory care units who completed the job plan item between 2021 and 2023 were analyzed. Frequencies and counts using individual participant responses were calculated for each job plan category and for reasons for intending to leave, with all results calculated separately for 1-year and 3-year time horizons across the study period (2021 to 2023). In addition, the top five most frequently reported specific reasons for intending to leave were identified and summarized for each year and time horizon.

To examine differences in work environment over time, Welch one-way ANOVAs were conducted to compare mean PES scores and all subscale scores across the survey years. When statistically significant differences were detected, post hoc tests were conducted to identify pairwise differences between survey years. To explore differences in job plans and intent-to-leave reasons by survey year, chi-square tests were conducted separately for 1-year and 3-year time horizons, comparing survey year with job plan categories (stay in current position, stay but change position, leave nursing) and intent-to-leave categories (work-related dissatisfaction, change in nursing career, home/personal life reasons).

## RESULTS

### Description of the sample

A sample description table provides a breakdown of the number of participating units, hospitals, and unit types included each year (see Table 1). On average, ambulatory care nurses are 43 years of age and most hold a Bachelor of Science in Nursing (BSN) as their highest degree.

**TABLE 1:** Individual RN-level and unit-level sample characteristics

	2021		2022		2023	
**Individual RN-level sample**						
	**n**		**n**		**n**	
Participants	16,406		18,787		17,105	
Units	1,921		2,320		2,041	
Hospitals	220		300		281	
***Number of units by unit type***						
Cancer Care	274		325		291	
Cardiac Services	142		218		180	
Endoscopy	155		211		184	
Interventional Cardiology/Radiology	261		356		318	
Outpatient Clinics	21		20		13	
Primary Care	218		192		166	
Procedural Unit	242		352		314	
Short Stay/Observation	102		137		120	
Specialty Care	468		483		416	
Urgent Care	38		26		39	
***RN characteristics***						
Education	n	%	n	%	n	%
Diploma	474	2.91	423	2.27	387	2.29
Associates	2,823	17.35	3,072	16.50	3,254	19.21
Baccalaureate	11,064	68.00	12,964	69.63	11,510	67.97
Masters	1,800	11.06	2,047	10.99	1,689	9.97
Doctorate	110	0.68	113	0.61	95	0.56
	Mean	SD	Mean	SD	Mean	SD
Age	44.29	11.54	43.60	11.59	43.61	11.68
**Unit-level sample**						
	n		n		n	
Units∗	1,263		1,423		1,277	
Hospitals	200		260		246	
***Number of units by unit type***						
Cancer Care	189		213		191	
Cardiac Services	76		98		83	
Endoscopy	123		142		139	
Interventional Cardiology/Radiology	199		235		206	
Outpatient Clinics	4		7		5	
Primary Care	143		120		91	
Procedural Unit	151		206		191	

∗ Only units with 5 or more responses were included in the sample.

### Practice environment

**FIGURE 1:**
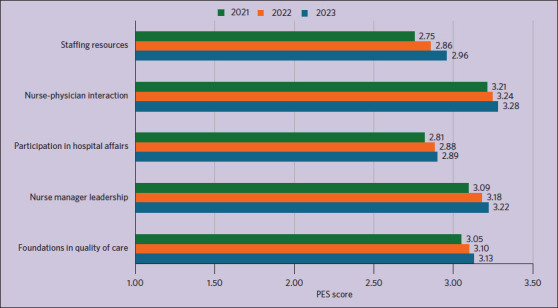
PES subscale scores by survey year

Descriptive statistics revealed an increase in the mean PES scores and all subscales over the 3 years, with mean PES score rising from 2.99 in 2021 to 3.05 in 2022 and further to 3.10 in 2023 (see Figure 1 for subscale scores). Welch's ANOVA revealed statistically significant differences in mean PES scores across survey years, *F*(2, 1956.39) = 32.60, *P* < .001, as well as across all subscales: staffing resources, *F*(2, 2543.11) = 71.38, *P* < .001; nurse leadership and support, *F*(2, 1960.60) = 25.39, *P* < .001; foundations in quality of care, *F*(2, 2540.29) = 24.81, *P* < .001; nurse-physician interaction, *F*(2, 1952.26) = 11.74, *P* < .001; and nurse participation in hospital affairs, *F*(2, 1967.02) = 13.77, *P* < .001.

Following the significant results of the ANOVA, post hoc comparisons revealed consistent patterns across the mean PES score and all subscales, with significantly lower scores in 2021 compared with both 2022 and 2023. The only exception was the nurse-physician interaction subscale, where 2021 scores were significantly lower than 2023 but not significantly different from 2022. These findings suggest an improvement in nurses' perceptions of the practice environment across domains beginning in 2022, with scores remaining elevated in 2023 compared with 2021.

### Job plans

**FIGURE 2:**
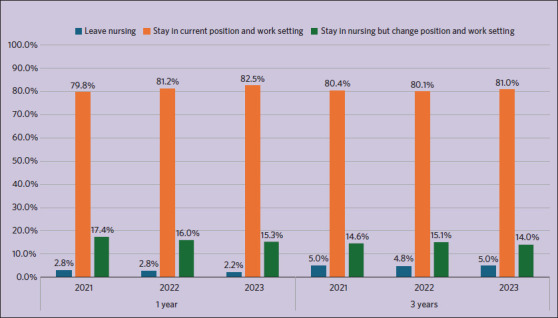
Percentage of nurses' job plans by survey year for 1-year and 3-year time horizons

A chi-square test assessing the relationship between survey year and nurses' 1-year job plans was statistically significant (χ^2^ = 44.50, *P* < .001), indicating career plans varied significantly across all 3 years (see Figure 2). The percentage of nurses planning to leave nursing was consistent at 2.8% in both 2021 and 2022, followed by a decrease to 2.2% in 2023. The proportion of nurses intending to stay in their current position and work setting increased from 79.8% in 2021 to 81.2% in 2022 and further to 82.5% in 2023. Conversely, the percentage of nurses planning to stay in nursing but change their position gradually declined from 17.4% in 2021 to 16.0% in 2022 and further to 15.3% in 2023. These findings suggest a trend of increasing retention over time, particularly in 2023, alongside a modest decline in turnover retentions.

In contrast, a chi-square test examining the relationship between survey year and nurses' 3-year job plans didn't reveal a statistically significant association (χ^2^ = 6.79, *P* = 0.147). The proportion of nurses planning to leave nursing remained low across all 3 years (ranging from 4.8% to 5.0%), whereas the majority consistently indicated plans to stay in their current position (80.4% to 81.0%) with minimal variation (see Figure 2). These results suggest that, although short-term career intentions showed more variation, long-term career plans were stable across the survey's 3 years.

### Intent to leave

**FIGURE 3:**
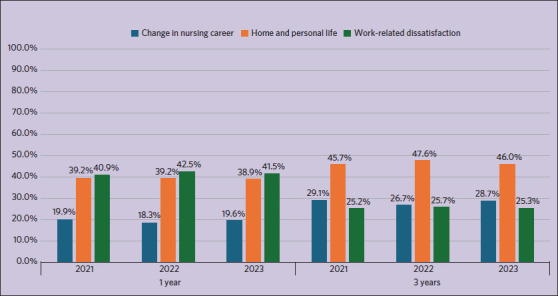
Percentage of intent-to-leave categories by survey year for 1-year and 3-year time horizons

***Primary categories for intent to leave***. A chi-square test examining the relationship between survey year and primary categories for intent to leave within 1 year didn't reveal a statistically significant association (χ^2^ = 2.96, *P* = .565), suggesting the reasons for intent to leave remained relatively stable over the 3 years (see Figure 3). Work-related dissatisfaction was reported the most frequently across the 3 years (ranging from 40.9% to 42.5%), followed by home and personal life reasons (ranging from 38.9% to 39.2%). Nursing career change was the least cited reason (ranging from 18.3% to 19.9%).

A chi-square test examining the relationship between survey year and primary categories for intent to leave within 3 years also didn't reveal a statistically significant association (χ^2^ = 3.40, *P* = .494). Home and personal life factors were the most frequently reported across the 3 years (ranging from 45.7% to 47.6%), followed by change in nursing career (ranging from 26.7% to 29.1%). Work-related dissatisfaction was the least cited reason (ranging from 25.2% to 25.7%).

***Top cited individual reasons for intent to leave***. At the 1-year time horizon, the most frequently reported specific reasons for intending to leave included home and personal life, compensation and pay, staffing or workload issues, and family obligations. At the 3-year horizon, home and personal life and family obligation continued to be prominent, and reasons related to nursing education—including pursuing further education and advancing in a specialty—were reported more frequently. These specific reasons for intending to leave remained consistent across survey years, emphasizing the consistent interplay of personal, financial, and professional development factors in nurses' short-term and long-term career planning.

## DISCUSSION

Overall, the results of this study indicate a positive upward trend of ambulatory nurses' perceptions of their practice environment. One exception is noted: the nurse-physician interaction subscale didn't have the same positive trajectory. Evidence demonstrates that positive nurse-physician relationships contribute to quality patient care, lower mortality rates, and increased patient satisfaction.^12^ Strong interprofessional relationships are critical to support newer team-based care models and have demonstrated positive patient outcomes as well as increased job satisfaction.^13^

Our analysis revealed that negative nurse--physician interactions were reported more often than positive relationships. This finding suggests nurse leaders should employ strategies to build collaborative cultures that promote team-based care, open communication, and mutual respect to achieve better quality care, safety, and patient experience. Nurse leaders can explore the implementation of programs like TeamSTEPPS from the Agency for Healthcare Research and Quality (AHRQ) to promote positive teamwork and communication. Krivanek and colleagues found that clarifying role and responsibilities through AHRQ's TeamSTEPPS framework enhanced nurse perceptions of improved teamwork, promoted a culture of safety, and increased workplace efficiency, all contributing to improving workplace satisfaction.^14^

Ambulatory care nurses in our study reported a lower intent to leave (18%) in comparison to about 30% for nurses working in other settings.^15^ These findings suggest ambulatory care nurses find their work and careers meaningful and satisfying. With the knowledge that intent to leave is lower for ambulatory care nurses, nurse leaders can maintain a level of work satisfaction by remaining vigilant to assess if their current environment becomes increasingly complex, adding to their employees' work burden.^16^ Increased patient complexity can negatively impact the ambulatory workforce's capacity to provide quality care and be a possible driver for ambulatory nurses' intent to leave. Thus, as shifts occur in the ambulatory care environment, nurse leaders should regularly engage in workforce planning to optimize the skill mix and roles of their team members to support high-quality care and maintain strong employee engagement.^17^-^19^

Interestingly, in this study, nurses cited factors in their personal environment—such as family obligations, relocation, or a more desirable commute—as their main reasons for leaving, versus workplace-related factors. This finding contrasts with Friese and colleagues who identified workplace-related factors, such as emotional strain, compensation, patient ratios, staffing concerns, and lack of support, as key predictors of intent to leave for nurses in general, not specifically within ambulatory care settings.^20^ Our study findings were different in that intent to leave was more associated with factors in nurse's personal and home environment than work-related factors, such as pay and compensation or staff/workload. Although factors in a nurse's home and personal environment were cited as reasons to leave, pay and compensation were reported as the second-most common reason in years 2 and 3; thus, continued evaluation of fair compensation may be important to maintain experienced staff over time.

Future job and career planning increased at year 3 of this study, indicating that nurse leaders can anticipate as their workforce becomes more experienced in their roles, many may be planning to advance their careers through further education or professional development. Nurse leaders can support these nurses by providing opportunities to remain at work while returning to school or pursuing professional development through certifications, such as the ambulatory care nursing certification. Such opportunities can help nurses validate their clinical knowledge and skills and develop professionally while remaining in ambulatory care nursing.

Collectively, these findings reflect that intent-to-leave patterns for short- and long-term career plans are mostly positive. Although work-related dissatisfaction was the most prominent reason for 1-year intent to leave, home and personal life factors were more influential in the 3-year time frame, suggesting a shift in priorities as career timelines extended. Nurse leaders should implement strategies that promote flexibility and adaptability and make room for nurses to attain their career goals of further education or advancement at their workplace.

## LIMITATIONS

One of the main limitations of this research is the use of a secondary data set. As a result, the data reported only represents settings that administer the PES-NWI survey to their ambulatory nursing workforce. Furthermore, many organizations who participate in this annually administered survey are on the Magnet journey or have received Magnet recognition. As a result, these findings may not reflect the broader ambulatory care nursing workforce.

A second limitation pertains to survey results excluded from the dataset when fewer than five respondents complete the survey from a clinic or department to protect anonymity. Thus, this survey procedure may disproportionately exclude the perspectives of nurses in rural or other small ambulatory clinic settings. Finally, only direct care nurses are eligible to participate in the survey, excluding the perspectives of nurses who practice in ambulatory care settings in other capacities, such as professional development or administrative roles. Nurses who practice in these roles also contribute to and are impacted by the health of the work environment.

## IMPLICATIONS FOR NURSE LEADERS

The findings of this research reinforce the importance of routinely assessing the health of the work environment where ambulatory nurses practice. Understanding nurse perceptions about various facets of the work environment is essential to appreciate what is working well and what needs to be improved to enhance joy at work and the overall nurse experience.^21^ Nurse leaders have a golden opportunity to discuss survey scores with frontline nurses to gain clarity about what's occurring in the practice environment—for instance, in staffing and resources or foundations of quality of care—as well as explore the career intentions of their staff.

Through close collaboration with their teams, nurse leaders can identify key opportunities for improvement, devise specific strategies to improve these facets of the practice environment, and ensure these factors are integrated into retention plans across ambulatory care settings. For instance, Snyder and colleagues implemented “stay interviews” to discover the existing factors keeping their nurses engaged and motivated, as well as learn how the work environment could be improved to maximize retention and enhance overall nurse satisfaction.^22^ The impact of any implemented strategies can be assessed through routine assessments of the nursing workforce. The PES-NWI survey or other validated tools—such as the Essentials of Magnetism II, the Healthy Work Environment Inventory, or the Healthy Work Environment Survey—could be used.^9^,^23^-^25^

As an exemplar, one innovative approach that nurse leaders at the VA Portland Health Care System (VAPORHCS) used to improve the nursing practice environment was a rapid-process improvement event (VAPORHCS, unpublished Nursing Annual Report, 2018). In this 3-day event, 80 frontline nurses and nurse leaders across the system gathered to understand the current environment by first reviewing survey scores. They examined root causes of low-scoring domains from the perspectives of nurses in diverse clinical settings. These root causes helped nurses and leaders generate hypotheses to implement mini-PDSA (Plan-Do-Study-Act) changes in their units or clinics.

For example, one practice change involved sending regular communications about quality improvement and evidence-based practice initiatives to ensure staff were aware of how nurses were leading practice improvements that positively impacted clinical outcomes. Another change nurses tested involved a toolkit detailing available professional development opportunities for nurses to advance their clinical and leadership competencies. Collectively, these strategies resulted in improvements in the next practice environment survey, where many domains outperformed national benchmarks (Halm, personal communication, 2024). Ambulatory care nursing leaders could modify and use this rapid process improvement model to collaborate with their teams to improve the practice environment.

## OPTIMIZING PRACTICE ENVIRONMENTS

This study reported intent to leave and future job/career planning for nurses in ambulatory settings over a period of 3 years, illuminating a nurse's view of their intentions from their first year to their third year on the job. Findings show that nurses in ambulatory care settings are more influenced to leave the practice environment due in part to personal factors than factors related to the work environment with one noted exception: the nurse-physician relationship. These participants reported negative nurse-physician relationships more often in years 2 and 3, offering a focused opportunity for nursing leadership to maintain or create positive practice environments as a method of improving retention and job satisfaction for ambulatory care nurses.

This study also found that, as ambulatory care nurses gain time and experience in their setting, their desire to advance their careers increases, giving nurse leaders an opportunity to create flexible and supportive environments to return to school or dedicate time to work toward ambulatory care nurse certification while staying in their current settings.

Nurses are more likely to stay in work settings where they have a voice in nursing practice, work in respectful environments, and have strong nursing leadership. Ambulatory nursing leaders can use this study to better understand factors nurses are navigating to stay in their current settings, as well as recognize the ways nurses' needs evolve over time. Nurse leaders can also use this study to enhance teamwork and improve communication skills between the interprofessional roles needed to effectively and efficiently care for patients and family members with increasing complex care and needs. Ultimately, better practice environments for nurses means better patient experiences.
